# Treatment approaches to patients with multiple sclerosis and coexisting psoriasis – A longitudinal multicenter observational cohort study

**DOI:** 10.1016/j.neurot.2026.e00914

**Published:** 2026-04-29

**Authors:** Tobias Brummer, Saskia Räuber, Jasmin Jakob, Alice G. Willison, Muriel Schraad, Maria Protopapa, Maria Isabel Fleischer, Joshua M. Boeckers, Tobias Ruck, Sven G. Meuth, Frauke Zipp, Vinzenz Fleischer, Stefan Bittner

**Affiliations:** a Department of Neurology, Research Center for Immunotherapy (FZI) and Focus Program Translational Neuroscience (FTN), Rhine Main Neuroscience Network (rmn2), University Medical Center of the Johannes Gutenberg University Mainz, Mainz, Germany; b Department of Neurology, Medical Faculty and University Hospital Düsseldorf, Heinrich Heine University of Düsseldorf, Düsseldorf, Germany; c Department of Dermatology, University Medical Center of the Johannes, Gutenberg-University Mainz, Mainz, Germany; d Department of Neurology, Ruhr University Bochum, BG University Hospital Bergmannsheil, Bochum, Germany

**Keywords:** Multiple sclerosis, Psoriasis, Comorbidities, Immunotherapy

## Abstract

Neurologists frequently encounter multiple sclerosis (MS) patients with coexisting autoimmune disorders such as psoriasis (PsO). The evolving landscape of immunotherapies has made treatment decisions more complex, yet also opened opportunities for therapies targeting shared immunopathogenic mechanisms. However, evidence for this patient group remains limited to case reports and small series. In this multicenter observational study, 420 MS patients were screened, identifying 38 with PsO. These were compared with MS-only patients regarding demographics, disease course, Expanded Disability Status Scale (EDSS), and disease-modifying therapy (DMT) use. DMT histories, reasons for discontinuation, and adverse events were analyzed. Logistic regression assessed PsO and MS worsening, using dimethyl fumarate (DMF) as reference. MS/PsO patients were older at diagnosis (37.6 vs. 33.0 years). EDSS and disease course showed no statistically significant differences. DMF was most frequently used (68%), with low rates of PsO (8%) and MS worsening (15%). PsO worsening was common under teriflunomide (75%), interferons (38%), and sphingosine-1-phosphate (S1P) modulators (40%). Logistic regression suggested higher odds under teriflunomide (OR = 16.5, *p = 0.028*), interferons (OR = 16.5, *p = 0.004*), and S1P-modulators (OR = 8.25, *p = 0.047*). Anti-CD20 therapies (OR = 2.75, *p = 0.257*) and natalizumab (OR = 1.83, *p = 0.635*) showed non-significant trends. Interleukin-17 (IL-17) inhibitors were well tolerated, with >50% stable in both conditions and no significant MS worsening (OR = 1.67, *p = 0.546*). IL-17 inhibitors and DMF showed a favorable tolerability in MS/PsO. Teriflunomide, interferons, and S1P-modulators frequently exacerbate PsO. CD20 therapies and natalizumab remain effective for MS but may worsen PsO; IL-17–based combinations appear promising in highly active disease.

## Graphical abstract

**Figure:**
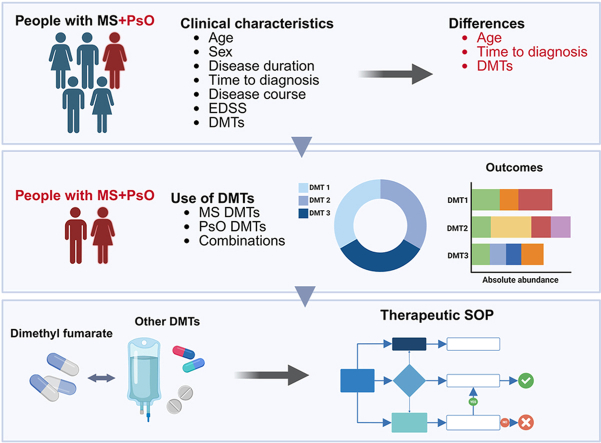

## Introduction

Multiple sclerosis (MS) and psoriasis (PsO) are chronic inflammatory disorders that, while affecting different organ systems, share a significant immunopathogenic overlap [1]. MS is characterized by autoimmune-mediated demyelination and neurodegeneration within the central nervous system [2], whereas PsO manifests as a chronic inflammatory skin condition with erythematous, scaly plaques [3]. Both conditions involve dysregulation of T-helper 17 (Th17) cells and share genetic risk factors, including polymorphisms in the interleukin-23/IL17 network [1,3], suggesting common inflammatory pathways. Furthermore, recent population-based studies have identified an increased risk of PsO among patients with MS [4]. Despite these shared mechanisms, the co-occurrence of MS and PsO remains relatively rare, and the clinical management of patients affected by both conditions is particularly challenging [5,6]. Several treatments effective for one disease may adversely impact the other. For instance, interferon-beta — widely used in MS — has been associated with PsO flares in different case reports [7,8]. Conversely, certain agents such as dimethyl fumarate and interleukin-17 (IL-17) antibodies have shown promise in treating both diseases [[9], [10], [11]], likely due to their effects on shared immunological targets. However, not all immunotherapies offer cross-disease benefit. Notably, anti-IL-23 therapies, despite their efficacy in PsO, have not demonstrated benefit in MS [12], reflecting differences in disease-specific pathogenesis. Furthermore, a growing area of concern involves the use of CD20-targeting therapies, such as ocrelizumab or ofatumumab, which are effective in controlling MS disease activity. However, emerging reports suggest these therapies may worsen or trigger PsO in some patients [[13], [14], [15], [16], [17]], raising important safety considerations for their use in individuals with comorbid PsO. Despite these important clinical observations, robust data on the management of patients with coexisting MS and PsO are lacking and the current body of evidence is limited to isolated case reports and small case series [5,18], with no larger systematic studies available to guide treatment decisions. This lack of data leaves clinicians navigating complex therapeutic decisions with limited evidence from the literature.

This study seeks to address this gap by systematically evaluating the clinical characteristics, treatment responses, and safety profiles of disease-modifying therapies (DMTs) in a cohort of patients with both MS and PsO or psoriatic arthritis (PsA). By comparing this cohort to a population of MS patients without PsO, we aim to identify treatment strategies that optimize outcomes for patients with both conditions while minimizing the risk of disease exacerbation. Our findings offer valuable insights into the safe and effective management of this unique and underserved patient population.

## Materials and methods

### Study design and patient cohort

This retrospective, observational cohort study was conducted at two German tertiary MS centers. The primary study population comprised 420 multicentric patients diagnosed with multiple sclerosis (MS) who were screened for coexisting diagnoses of psoriasis (PsO) or psoriatic arthritis (PsA) through detailed electronic health record review. Screening for PsO/PsA was based on systematic review of diagnostic codes, physician correspondence, medication histories (including dermatologic and rheumatologic agents), and routinely documented comorbidities recorded during clinical visits. Diagnoses were confirmed by dermatology and/or rheumatology specialists where applicable, while patients without documented evidence were classified as negative. No systematic dermatologic or rheumatologic screening was performed across the entire cohort. Patients were included between 2010 and 2025 as part of an in-house, prospectively maintained observational MS cohort. Inclusion in this cohort required written informed consent and the availability of standardized longitudinal clinical and treatment data. Inclusion was therefore restricted to patients with sufficient follow-up to allow reliable assessment of treatment courses and outcomes. Consequently, the study population represents a subset of all MS patients treated at both centers.

All patients met the 2017 McDonald criteria for MS diagnosis and had at least one year of clinical follow-up data. Baseline was defined as the first documented clinical assessment at the participating centers within the observation period, at which standardized clinical variables (including Expanded Disability Status Scale [EDSS], disease course, and treatment status) were available. Given the retrospective observational design, the study was not registered.

### Clinical assessment

Patients with both MS and PsO/PsA (MS/PsO group) were compared to MS patients without PsO (MS-only group). Demographic and clinical variables were extracted, including age, sex, disease duration, MS disease course (relapsing-remitting, secondary progressive, or primary progressive), and EDSS scores. The EDSS score was recorded at the first visit and at the most recent follow-up.

### Therapy classification and analysis

A comprehensive evaluation of DMTs was performed for all patients. Current and previous DMTs were recorded, along with treatment duration, reasons for discontinuation, and adverse events. Therapies were grouped by mechanism of action, and particular attention was paid to DMTs commonly used in either MS and/or PsO, including fumarates, interferons, glatiramer acetate, sphingosine-1-phosphate (S1P) receptor modulators, anti-CD20 monoclonal antibodies, and IL-17 or IL-23 inhibitors. Analyses of treatment-specific outcomes in the MS/PsO cohort were performed at the level of DMT exposures, reflecting the longitudinal and sequential nature of treatment in this population, whereby individual patients could contribute multiple treatment episodes. No formal repeated-measures correction was applied due to the small cohort size, sparse event counts, and unbalanced treatment-class distribution. A minimum DMT exposure duration of 6 months was defined a priori to ensure sufficient treatment exposure for attribution of MS or PsO worsening. Importantly, no patients with MS/PsO or DMT exposures were excluded based on treatment duration. Due to small agent-specific numbers, IL-17 inhibitors and anti-CD20 monoclonal antibodies were analyzed as therapeutic classes rather than individual agents. The frequency and nature of PsO exacerbation or improvement during each therapy were documented based on patient-reported outcomes, clinical assessments, and/or dermatological records.

### Definition of disease activity

MS progression was defined as any documented sign of disease activity, including clinical relapses, radiological disease activity, relapse-associated worsening (RAW) or progression independent of relapse activity (PIRA), in accordance with current consensus definitions [19,20]. Due to the small MS/PsO cohort and expected low event rates for individual components, a combined MS-worsening endpoint was used to maintain sensitivity. Standardized PsO severity scores (e.g., PASI or body surface area [BSA]) were not consistently available because of decentralized dermatologic care and the retrospective study design; PsO worsening was therefore defined based on patient-reported symptoms and clinician-documented flare-ups, new or expanding lesions, and/or escalation of topical or systemic psoriasis therapy as recorded in dermatology or neurology records, without application of a predefined PASI or BSA cut-off.

### Statistical analysis

Descriptive statistics were used to summarize demographic and clinical characteristics. Normality of continuous variables was assessed using the Shapiro–Wilk test. Between-group comparisons were performed using Mann-Whitney *U* test for continuous variables and Chi-square test or Fisher's exact test for categorical variables. Logistic regression analyses were performed to compare the odds of PsO worsening and MS worsening across different DMTs. DMF was used as the reference comparator, given its approval for both MS and PsO, its frequent use in this cohort, and its low PsO exacerbation rates. To account for multiple testing across baseline characteristics, Benjamini–Hochberg false discovery rate (FDR) correction was applied to the set of baseline p-values, and adjusted p-values (q-values) are reported. Formal multiple-testing correction was not applied to treatment-related outcome analyses due to non-independent sequential DMT exposures, sparse event counts, and the exploratory nature of these analyses; nominal p-values are therefore reported. Due to low event rates and small group sizes for some therapies, descriptive statistics were reported where regression analysis was not feasible. A p-value of <0.05 was considered statistically significant. All analyses were conducted using SPSS (version 29.0; IBM Corp., Armonk, NY) and GraphPad Prism 10.

## Results

### Patient characteristics

**Fig. 1:**
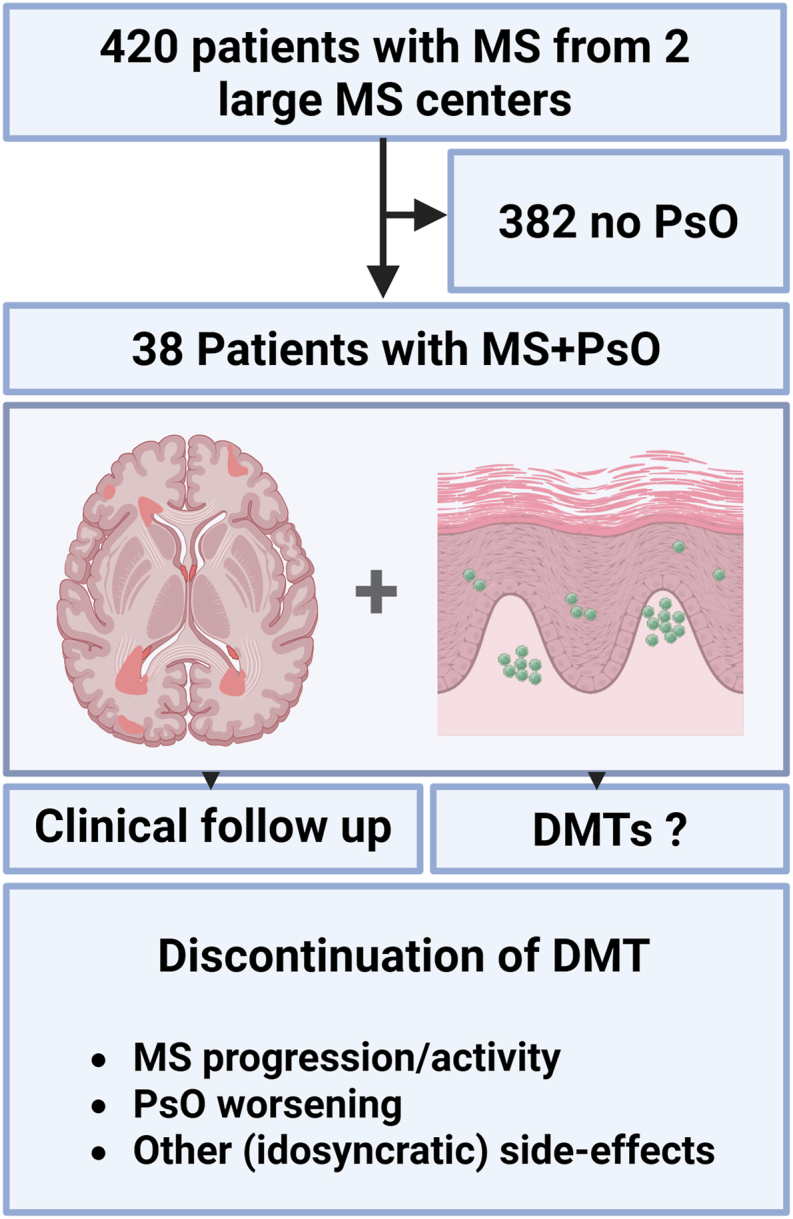
**Study analysis design. DMT:** disease modifying therapy; **MS:** multiple sclerosis; **PsO:** psoriasis.

A total of 420 patients were included, comprising 382 with MS without psoriasis (PsO) and 38 with coexisting MS and PsO, and a mean clinical follow up of 6 ± 4 years (Fig. 1). Notably, 80% of MS/PsO patients had psoriasis onset prior to MS diagnosis. Patients with MS and PsO were significantly older at the time of data collection (mean 45.4 ± 11.9 years) compared to the MS-only cohort (mean 40.3 ± 11.8 years, *p = 0.014, adj. p = 0.054*) and were also older at diagnosis (mean 37.6 ± 11.7 vs. 33.0 ± 10.7 years, *p = 0.021, adj. p = 0.056*). There were no significant differences regarding age at first event, sex distribution, disease duration, EDSS, or disease course between groups (Table 1). Notably, current DMT use differed significantly between groups (*p < 0.0001, adj. p = 0.0008*). In the MS + PsO cohort, 11% were untreated, 32% were on moderate efficacy therapies, 37% on high efficacy therapies, and 21% on other treatments, while in the MS-only cohort, 20% were untreated, 53% were on moderate therapies, 27% on high efficacy therapies, and less than 1% on other treatments (Table 1). Within the MS/PsO cohort, six patients (15.8%) had a diagnosis of psoriatic arthritis (PsA), while the remaining patients had cutaneous PsO only; a descriptive comparison of MS + PsO and MS + PsA patients is provided in Table S1. Patients with PsA were slightly younger and had shorter disease durations than PsO patients. Notably, most PsA patients were treated with IL-17 inhibitors (83%), and most had prior exposure to TNFα-blocking therapy before MS diagnosis (83%). To assess whether observed baseline patterns reflect psoriasis-specific rather than general autoimmune comorbidity features, baseline characteristics were additionally compared with MS patients with inflammatory bowel disease or rheumatoid arthritis, revealing no significant differences (Table S2).

**Table 1:** Patient characteristics.

	MS cohort (n = 382)	MS + PsO (n = 38)	*p-value*	*adj. p-value*^*c*^
Age (years) [mean, SD]	40.26 ± 11.80	45.39 ± 11.85	***0.0135*^*a*^**	*0.0540*
Age at diagnosis (years) [mean, SD]	33.04 ± 10.72	37.58 ± 11.69	***0.0211*^*a*^**	*0.0563*
Age at first event (years) [mean, SD]	31.82 ± 10.53	34.94 ± 10.53	*0.1147*^*a*^	*0.2294*
Sex (female) [%]	262 (68.6)	24 (63.2)	*0.5843*^*b*^	*0.5843*
Disease duration (years) [mean, SD]	8.659 ± 16.27	9.297 ± 9.938	*0.5085*^*a*^	*0.5811*
Disease course, n (%)				
RRMS	340 (89)	31 (82)		
SPMS	24 (6)	5 (13)	*0.2748*^*b*^	*0.3664*
PPMS	18 (5)	2 (5)		
EDSS [mean, SD]	2.076 ± 1.881	2.526 ± 2.184	*0.2430*^*a*^	*0.3888*
Current DMT, n (%)				
None	77 (20)	4 (11)		
Moderate	202 (53)	12 (32)	***<0.0001*^*b*^**	***<0.0008***
High	102 (27)	14 (37)		
Other	1 (0)	8 (21)		

**Statistic tests: a:** Mann-Whitney *U* Test**, b:** Fisher exact or Chi-square Test, **c:** FDR-correction with the Benjamini Hochberg method. **DMT:** Disease modifying therapy **(moderate:** Interferons, glatiramer acetate, fumarates, teriflunomide; **high:** Natalizumab, anti-CD20 antibodies, cladribine, S1P modulators; **other:** Azathioprine, methotrexate, anti-IL17A antibodies, sulfasalazine)**; EDSS:** Expanded disability status scale, **MS:** Multiple sclerosis**, PsO:** Psoriasis; **RRMS:** Relapsing remitting multiple sclerosis; **SD:** Standard deviation; **SPMS:** Secondary progressive multiple sclerosis; **PPMS:** Primary progressive multiple sclerosis.

### Use of DMTs and adverse events

**Fig. 2:**
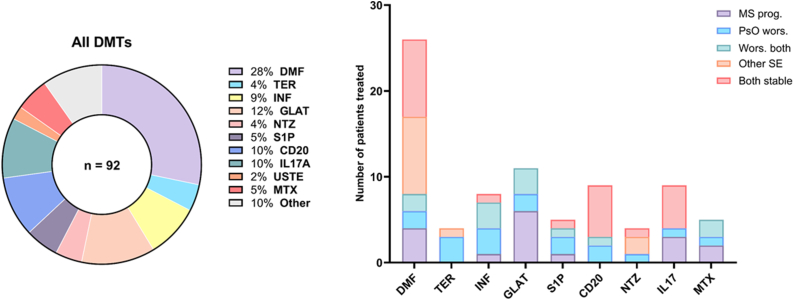
**Total number of DMTs in patients with MS/PsO (left) and reasons for DMT (dis)continuation (right). CD20:** anti-CD20 antibodies; **DMF:** dimethyl fumarate; **DMT:** disease modifying therapy; **GLAT:** glatiramer acetate; **INF:** interferons; **IL17A:** anti-IL17A antibodies; **MS:** multiple sclerosis; MS prog: MS progression (relapse associated worsening or progression independent of relapse activity) **MTX:** methotrexate; **NTZ:** natalizumab; **PsO:** psoriasis; PsO wors.: PsO worsening. SE: Side-effects; **S1P:** S1P-modulators; **TER:** Teriflunomide; **USTE:** ustekinumab. **Other:** azathioprine, cladribine, cyclosporine A, daclizumab, mitoxantrone, sulfasalazine.

In the MS + PsO cohort, a total of 92 DMT courses were documented, reflecting multiple treatments per patient (2.4 DMTs per patient) (Fig. 2). Dimethyl fumarate (DMF) was the most frequently used DMT (68.4% of patients), followed by glatiramer acetate (GLAT, 28.9%), CD20 therapies (23.7%), IL-17A antibodies (23.7%) and interferons (INF, 21.1%). Other therapies, including teriflunomide (TER), natalizumab (NTZ), sphingosine-1-phosphate (S1P) modulators, methotrexate (MTX), ustekinumab (USTE), and additional agents (sulfasalazine, cladribine, mitoxantrone, daclizumab, cyclosporine A, cyclophosphamide), were used less frequently within this cohort.

Overall, MS worsening occurred in 26.1% of DMT exposures, with the highest rates under “other” therapies (77.8%) and GLAT (54.5%). Notably, MS worsening occurred in 33.3% of IL-17A treatment courses. DMF was associated with a lower rate of MS worsening (15.4%), while no MS worsening was observed under TER, NTZ, or CD20 therapies during the observation period. PsO worsening was observed in 20.7% of DMT exposures, with the highest rates under TER (75%), INF (37.5%), and S1P modulators (40%). DMF was associated with the lowest PsO worsening rate (7.7%), followed by IL-17 inhibitors (11.1%). Both CD20 monoclonal antibodies and natalizumab were associated with intermediate rates of PsO worsening, at 22% and 25%, respectively. Worsening of both MS and PsO simultaneously occurred in 15.2% of DMT exposures, most frequently under INF (37.5%), GLAT (27.3%), and MTX (40%), while rare under DMF (7.7%) NTZ and IL17A antibodies. No MS worsening was recorded only under NTZ and TER (Fig. 2). However, the one patient who experienced worsening of both MS and PsO under a CD20 therapy had a primary progressive MS disease course. To assess whether coexisting PsO influences MS treatment response, we performed a descriptive comparison of MS worsening rates across the most common shared DMTs (INF, GLAT, DMF, and CD20 therapies) between the MS-only and MS/PsO cohorts. MS worsening rates and baseline age and sex distributions were comparable across DMT classes with no statistically significant differences detected (Table S3).

Other side effects unrelated to MS or PsO worsening occurred in 50% of NTZ, 35% of DMF, and 25% of TER treatments. DMF was primarily discontinued due to lymphopenia and gastrointestinal symptoms, while natalizumab was discontinued in all cases due to JCV seroconversion. Importantly, 7 patients (18.4% of the MS + PsO cohort) experienced their first demyelinating event under treatment with a TNFα-blocker. Notably, three patients received a combination therapy with the IL17A-antibody secukinumab (SEK) and CD20 monoclonals without experiencing any adverse events or disease worsening.

### Therapy-specific risk of MS and PsO worsening

To compare the risk of PsO worsening across commonly used MS DMTs and corroborate the results from the descriptive data, we performed exploratory logistic regression analyses using DMF as the reference (Table 2), as it is approved for both MS and PsO, frequently used in this cohort, and associated with low PsO exacerbation rates. TER and INF were significantly associated with a 16.5-fold higher odds of PsO worsening (TER: OR = 16.5, 95% CI: 1.35–201.3, *p = 0.028, INF:* OR = 16.5, 95% CI: 2.41–112.8, *p = 0.004*). S1P modulators also showed increased odds (OR = 8.25, 95% CI: 1.03–66.19, *p = 0.047*), while CD20 therapies and natalizumab showed elevated but non-significant odds compared to DMF (ORs: 2.75 and 1.83; *p = 0.257 and 0.635*). GLAT also showed a higher odds of PsO worsening, however this was not statistically significant (OR = 4.58, 95% CI: 0.93–22.58, *p = 0.061*). Baseline characteristics differed systematically across MS-DMT groups (Table S4), with higher-efficacy therapies used in older patients with higher EDSS and more progressive disease. In contrast, IL-17A inhibitors and DMF showed similar baseline profiles, supporting a more direct comparison. Accordingly, analyses of MS worsening across DMT classes were interpreted descriptively. Logistic regression comparing IL-17A inhibitors to DMF showed a non-significant 1.67-fold higher odds of MS worsening under IL-17A treatment (OR = 1.67, 95% CI: 0.32–8.76, *p = 0.546*). In a sensitivity analysis restricted to MRI-confirmed disease activity, logistic regression also showed no significant difference between IL-17A and DMF (OR = 2.10, 95% CI 0.39–11.43, *p = 0.391*), consistent with the composite MS endpoint. No MS worsening events occurred under ustekinumab (2 patients), precluding reliable regression analysis for this therapy.

**Table 2:** Logistic regression model.

Variable	Coefficient	*p-value*	Odds Ratio	95% CI
PsO worsening under MS DMTs				
Intercept (DMF)	−1.70	*0.002*	0.18	0.06–0.53
CD20 vs. DMF	1.01	*0.257*	2.75	0.48–15.79
NTZ vs. DMF	0.61	*0.635*	1.83	0.15–22.37
TER vs. DMF	**2.80**	***0.028***	**16.5**	**1.35**–**201.9**
S1P vs. DMF	**2.11**	***0.047***	**8.25**	**1.03**–**66.19**
INF vs. DMF	**2.80**	***0.004***	**16.5**	**2.41**–**112.8**
GLAT vs. DMF	1.52	*0.061*	4.583	0.93–22.58
MS worsening under PsO DMTs				
Intercept (DMF)	**−1.20**	**0.010**	**0.30**	**0.12**–**0.75**
IL17A vs. DMF	0.51	*0.546*	1.67	0.32–8.76

**CD20:** anti-CD20 antibodies; **DMF:** dimethyl fumarate; **DMT:** disease modifying therapy; **GLAT:** glatiramer acetate; **INF:** interferons; **IL17A:** anti-IL17A antibodies; **MS:** multiple sclerosis; **NTZ:** natalizumab; **PsO:** psoriasis; **S1P:** S1P-modulators; **TER:** Teriflunomide. The intercept represents the baseline odds of MS worsening under the reference category (DMF) within the model.

**Fig. 3:**
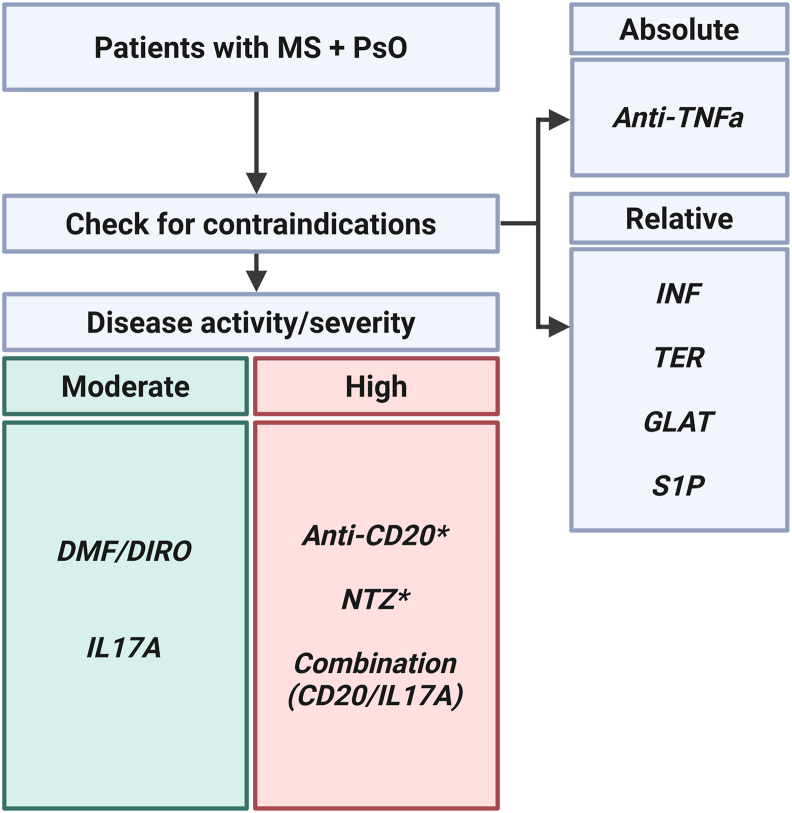
**Proposed clinical considerations for DMT selection in patients with MS/PsO. CD20:** anti-CD20 antibodies; **DIRO:** Diroxymelfumarate; **DMF:** dimethyl fumarate; **DMT:** disease modifying therapy; **GLAT:** glatiramer acetate; **High:** highly active MS. **INF:** interferons; **IL17A:** anti-IL17A antibodies; **Moderate:** moderately active MS. **MS:** multiple sclerosis; **NTZ:** natalizumab; **PsO:** psoriasis; **S1P:** S1P-modulators; **TER:** Teriflunomide. **∗** Careful risk-benefit evaluation. Suggestions include off-label use of drugs or non-approved combination therapies and potential side-effects should be observed carefully.

Eventually, we here – based on the generated dataset as well as a broad perspective on the literature - provide a proposed clinical decision-making flow chart to guide therapy selection in patients with MS and PsO based on our findings (Fig. 3).

## Discussion

In this study, we examined the clinical and therapeutic characteristics of patients with comorbid MS and PsO, an underexplored but clinically relevant co-occurrence. Our findings reveal significant differences in demographic and treatment profiles between MS/PsO patients and those with MS alone, offering novel insights into this dual autoimmune phenotype. Patients with both MS and PsO were generally older at analysis and at diagnosis but not at the first demyelinating event, suggesting a possible delay in MS diagnosis due to comorbidities and competing healthcare priorities [21]. Notably, while receiving different immunotherapies, these patients were not underrepresented in the use of highly active MS therapies, indicating they were not undertreated compared to other MS patients, despite possible concerns for PsO worsening. Notably, MS disease control was comparable between MS-only and MS/PsO cohorts, suggesting limited impact of PsO on MS-related treatment responses.

A particularly notable finding was that 18% of MS cases in the MS/PsO cohort were diagnosed after anti-TNFα therapy had been initiated for PsO or PsA, corroborating reports of anti-TNF-induced demyelination or unmasking of latent MS [[22], [23], [24]]. Anti-TNFα agents such as infliximab and etanercept have been implicated in demyelinating events, such as optic neuritis and MS onset, likely due to disruption of TNFα's dual role in immune modulation and neuroprotection [25].

Overall, DMF was the most frequently used DMT in our study, largely due to its dual indications and favorable immunologic profile [6,[26], [27], [28]]. Despite its clinical efficacy, many patients discontinued DMF due to treatment-limiting lymphopenia or gastrointestinal discomfort, highlighting its utility in patients with moderate disease activity provided hematologic monitoring is maintained. Surprisingly, S1P receptor modulators were associated with PsO exacerbation in a significant number of cases despite the evaluation of ponesimod in PsO trials [29,30]. However, there are also case reports documenting PsO exacerbations under S1P therapy with fingolimod, further supporting our findings [31]. This may be explained by altered S1P-mediated lymphocyte redistribution, particularly of skin-homing T cells, ultimately provoking psoriasiform flares. These findings challenge assumptions about S1P modulators' suitability in co-affected patients and suggest caution in MS patients with PsO history or active skin disease.

Many patients experienced PsO worsening while on teriflunomide or interferons. Teriflunomide, despite its convenience and efficacy in MS, may exacerbate PsO due to immune modulation inadvertently favoring psoriatic pathways, while interferons can activate type I interferon pathways implicated in psoriatic inflammation [7,8]. Although a positive study suggesting potential PsO benefits was performed with leflunomide [32], clinical experience [[33], [34], [35], [36], [37]] and our findings indicate a notable risk of exacerbation with its pro-drug teriflunomide. Similarly, a substantial proportion of patients on glatiramer acetate (GLAT) experienced PsO worsening, which may partly be explained by Koebner's phenomenon due to mechanical skin trauma at injection sites [7], warranting dermatologic consultation and close monitoring when using GLAT in this population. However, there is also evidence from animal experiments showing beneficial effects of GLAT in experimental PsO [38].

In contrast, IL17A antibodies emerged as one of the most promising options, with over half of patients remaining stable in both MS and PsO during treatment. This favorable profile aligns well with several case reports where the IL17A antibodies secukinumab or ixekizumab stabilized both conditions [9,11,[39], [40], [41]]. Notably, although secukinumab reached secondary endpoints in an MS trial, a randomized controlled trial of IL-17 inhibition in MS did not demonstrate primary efficacy. [11]. Together with previous reports and studies, our findings support the use of IL-17 blockers as potential monotherapy in MS/PsO patients with low to moderate disease activity, or as potential combination therapy with CD20 monoclonals or DMF [42,43] in patients with more active disease (Fig. 3). Furthermore, recent case based evidence also demonstrated favorable outcomes of IL-17 blockers with fingolimod or ofatumumab, supporting the plausibility of combination strategies in patients requiring high-efficacy MS treatment [[44], [45], [46]].

CD20 therapies indicated variable outcomes. A proportion of patients experienced PsO worsening on B-cell depleting agents, such as ofatumumab or ocrelizumab, aligning with previous reports of heterogeneous cutaneous responses [[13], [14], [15], [16], [17], [18],^47^]. However, logistic regression analysis revealed no significant increase in PsO worsening compared to patients treated with DMF. These discrepancies may relate to differences in disease stage, PsO severity, or concurrent therapies. Combination strategies using CD20 and IL-17 antibodies may represent a tolerable option in selected patients with highly active MS, balancing neurological disease control with psoriasis management (Fig. 3) [43]. Similarly, natalizumab, was also associated with psoriatic flare-ups in some patients. Although prior reports highlight natalizumab-induced skin disease [48,49], most of the MS/PsO patients in our cohort showed no PsO exacerbations. These inconsistencies suggest that patient selection and disease biology may modulate the risk of PsO worsening. Mechanistically, natalizumab generally increases peripheral lymphocyte counts by preventing their CNS trafficking [50], potentially driving lymphocyte redistribution, particularly of skin-homing T cells. However, as natalizumab is typically used in highly active MS, the risk of PsO exacerbation may be acceptable in favor of maintaining neurological stability.

To systematically evaluate treatment risks, we also performed logistic regression modeling using DMF as the reference, reflecting its approval for both MS and PsO and its low PsO exacerbation rates. This analysis suggested that teriflunomide, interferons, GLAT and S1P modulators were associated with higher odds of PsO worsening, while CD20 therapies and natalizumab showed a trend toward increased risk without clear significance. In a separate model, IL17A antibodies did not show a significant increase in MS worsening risk compared to DMF, while MS worsening under DMF remained low. Therefore, these findings support the use of DMF and IL17A antibodies as preferred first-line options for managing patients with coexisting MS and PsO (Fig. 3).

This study has several limitations inherent to the rarity of coexisting multiple sclerosis and psoriasis: The modest cohort size limited statistical power, precluded robust multivariable adjustment, and necessitated a primarily descriptive and hypothesis-generating analytical approach. Consequently, regression analyses should be interpreted as exploratory rather than confirmatory. In addition, many patients received multiple sequential disease-modifying therapies, preventing assignment of a single baseline disease severity per treatment class and limiting causal interpretation of treatment-associated outcomes. Analyses were therefore performed at the level of DMT exposures, which may have introduced non-independence of observations and potential bias related to prior treatment failure and disease severity. Baseline differences across DMT groups limited direct comparisons of MS worsening; accordingly, analyses were interpreted descriptively. In contrast, patients treated with IL-17A inhibitors and DMF showed similar baseline characteristics, supporting a more robust comparison. Although inflammatory disease activity and disability progression represent distinct pathological processes, the small number of events required the use of a combined MS worsening endpoint. The observational design is further subject to confounding by indication, as treatment selection was driven by disease severity, disease course, prior treatment response, and clinical judgment. Standardized psoriasis severity measures (e.g., PASI) were not consistently available due to decentralized dermatologic care, necessitating reliance on clinically documented worsening. No systematic dermatologic or rheumatologic screening was performed across the entire cohort; therefore, mild or subclinical cases may have been under detected. However, identified cases were specialist-confirmed and therefore likely represent clinically relevant disease. Finally, the study population represents a subset of patients with available longitudinal data from tertiary centers and may be biased toward individuals with more regular follow-up and more complete documentation. Despite these limitations, this represents the largest dataset to date and, to our knowledge, the only longitudinal evaluation of treatment-associated outcomes in patients with coexisting MS and PsO, providing clinically relevant real-world insights into this under-studied population.

Together, our findings reveal consistent treatment risk-benefit patterns in MS and PsO. Anti-TNFα therapies pose a high risk unmasking MS, necessitating avoidance in PsO patients at MS risk. Conversely, IL17A antibodies offer a favorable tolerability profile with low rates of documented PsO worsening, while teriflunomide and interferons frequently worsen PsO and should be used cautiously if at all in active PsO. Glatiramer acetate can exacerbate PsO, underscoring the need for dermatologic oversight even with platform therapies. CD20 therapies and natalizumab remain effective for MS but may exacerbate PsO, requiring a thorough risk-benefit evaluation, while combination strategies with IL17A antibodies may optimize disease control in this population.

## Informed consent

All patients included in this study had previously provided written informed consent for the use of their anonymized clinical data for research purposes as part of the respective institutional observational multiple sclerosis cohorts.

## Ethics approval

This study was approved by the local institutional ethics committees of the participating German tertiary multiple sclerosis centers and was conducted in accordance with the Declaration of Helsinki and its later amendments. Given the retrospective observational study design, the study was not registered.

## Author contributions

**TB:** Conceptualization; Methodology; Data curation; Formal analysis; Investigation; Methodology; Visualization; Writing – original draft. Writing – review & editing. **SR:** Data curation; Writing – review & editing. **JJ:** Data curation; Writing – review & editing. **AGW:** Data curation; Writing – review & editing. **MS:** Data curation; Writing – review & editing. **MP:** Data collection; Writing – review & editing. **MIF:** Writing – review & editing. **JMB:** Data curation; Writing – review & editing. **TR:** Resources; Writing – review & editing. **SGM:** Resources; Writing – review & editing. **FZ:** Conceptualization; Supervision; Funding acqusition; Resources; Writing – review & editing; **VF:** Conceptualization; Supervision; Funding acqusition; Resources; Writing – original draft; Writing – review & editing; **SB:** Conceptualization; Supervision; Funding acqusition; Resources; Writing – original draft; Writing – review & editing.

## Availability of data and material

The raw data used in preparation of the figures and tables will be shared in an anonymized format upon reasonable request by a qualified investigator for purposes of replicating procedures and results.

## Funding

This study was supported by the Deutsche Forschungsgemeinschaft (DFG; SFB CRC-TR-128 project number 213904703 to SGM, FZ, VF, and SB; SFB 1080 project number 221828878 and SFB CRC-1292 project number 318346496 to FZ; and SFB/TRR 355 project number 490846870 to SB) and the Hermann and Lilly Schilling Foundation (to SB).

## Declaration of competing interest

The authors declare the following financial interests/personal relationships which may be considered as potential competing interests: Saskia Raeuber reports a relationship with Merck Healthcare Germany GmbH that includes: consulting or advisory, speaking and lecture fees, and travel reimbursement. Saskia Raeuber reports a relationship with Bristol Myers Squibb that includes: travel reimbursement. Saskia Raeuber reports a relationship with Alexion Pharma Germany GmbH that includes: travel reimbursement. Saskia Raeuber reports a relationship with Roche Pharma AG that includes: speaking and lecture fees. Saskia Raeuber reports a relationship with Novartis Pharma GmbH that includes: funding grants. Saskia Raeuber reports a relationship with Sanofi-Aventis Deutschland GmbH that includes: funding grants. Tobias Ruck reports a relationship with Argenx Us, Inc. that includes: consulting or advisory, funding grants, speaking and lecture fees, and travel reimbursement. Tobias Ruck reports a relationship with Alexion that includes: consulting or advisory, funding grants, speaking and lecture fees, and travel reimbursement. Tobias Ruck reports a relationship with Celegene that includes: speaking and lecture fees and travel reimbursement. Tobias Ruck reports a relationship with Biogen that includes: consulting or advisory, funding grants, speaking and lecture fees, and travel reimbursement. Tobias Ruck reports a relationship with Roche that includes: consulting or advisory, funding grants, speaking and lecture fees, and travel reimbursement. Tobias Ruck reports a relationship with Sanofi that includes: consulting or advisory, funding grants, speaking and lecture fees, and travel reimbursement. Tobias Ruck reports a relationship with Novartis that includes: funding grants, speaking and lecture fees, and travel reimbursement. Tobias Ruck reports a relationship with Teva Pharmaceuticals USA Inc that includes: speaking and lecture fees and travel reimbursement. Tobias Ruck reports a relationship with Merck & Co Inc that includes: consulting or advisory and funding grants. Tobias Ruck reports a relationship with Johnson & Johnson MedTech that includes: consulting or advisory. Tobias Ruck reports a relationship with Immunovant Inc that includes: consulting or advisory. Tobias Ruck reports a relationship with SERB Pharmaceuticals that includes: funding grants. Sven G. Meuth reports a relationship with Argenx Us, Inc. that includes: travel reimbursement. Sven G. Meuth reports a relationship with Alexion that includes: funding grants, speaking and lecture fees, and travel reimbursement. Sven G. Meuth reports a relationship with Almirall that includes: funding grants, speaking and lecture fees, and travel reimbursement. Sven G. Meuth reports a relationship with Academy 2 that includes: speaking and lecture fees and travel reimbursement. Sven G. Meuth reports a relationship with Amicus Therapeutics Germany that includes: funding grants, speaking and lecture fees, and travel reimbursement. Sven G. Meuth reports a relationship with Bayer Health Care that includes: speaking and lecture fees and travel reimbursement. Sven G. Meuth reports a relationship with Biogen that includes: funding grants, speaking and lecture fees, and travel reimbursement. Sven G. Meuth reports a relationship with BioNTech that includes: speaking and lecture fees and travel reimbursement. Sven G. Meuth reports a relationship with Bristol Myers Squibb that includes: funding grants, speaking and lecture fees, and travel reimbursement. Sven G. Meuth reports a relationship with Datamed that includes: speaking and lecture fees and travel reimbursement. Sven G. Meuth reports a relationship with Demecan that includes: speaking and lecture fees and travel reimbursement. Sven G. Meuth reports a relationship with Desitin that includes: speaking and lecture fees and travel reimbursement. Sven G. Meuth reports a relationship with Diaplan that includes: speaking and lecture fees and travel reimbursement. Sven G. Meuth reports a relationship with DIU Dresden that includes: speaking and lecture fees and travel reimbursement. Sven G. Meuth reports a relationship with DPmed that includes: speaking and lecture fees and travel reimbursement. Sven G. Meuth reports a relationship with Gen Medicine and Healthcare Products that includes: speaking and lecture fees and travel reimbursement. Sven G. Meuth reports a relationship with Genzyme that includes: funding grants, speaking and lecture fees, and travel reimbursement. Sven G. Meuth reports a relationship with Hexal AG that includes: speaking and lecture fees and travel reimbursement. Sven G. Meuth reports a relationship with IGES that includes: speaking and lecture fees and travel reimbursement. Sven G. Meuth reports a relationship with Impulze GmbH that includes: speaking and lecture fees and travel reimbursement. Sven G. Meuth reports a relationship with Janssen Cilag that includes: speaking and lecture fees and travel reimbursement. Sven G. Meuth reports a relationship with KW Medipoint that includes: speaking and lecture fees and travel reimbursement. Sven G. Meuth reports a relationship with MedDay Pharmaceuticals that includes: speaking and lecture fees and travel reimbursement. Sven G. Meuth reports a relationship with Medudy that includes: speaking and lecture fees and travel reimbursement. Sven G. Meuth reports a relationship with Merck Serono that includes: funding grants, speaking and lecture fees, and travel reimbursement. Sven G. Meuth reports a relationship with MICE that includes: speaking and lecture fees and travel reimbursement. Sven G. Meuth reports a relationship with Mylan that includes: speaking and lecture fees and travel reimbursement. Sven G. Meuth reports a relationship with Neuraxpharm that includes: speaking and lecture fees and travel reimbursement. Sven G. Meuth reports a relationship with Neuropoint that includes: speaking and lecture fees and travel reimbursement. Sven G. Meuth reports a relationship with Novartis that includes: funding grants, speaking and lecture fees, and travel reimbursement. Sven G. Meuth reports a relationship with Novo Nordisk that includes: speaking and lecture fees and travel reimbursement. Sven G. Meuth reports a relationship with ONO Pharma that includes: funding grants, speaking and lecture fees, and travel reimbursement. Sven G. Meuth reports a relationship with Oxford PharmaGenesis that includes: speaking and lecture fees and travel reimbursement. Sven G. Meuth reports a relationship with QuintilesIMS that includes: speaking and lecture fees and travel reimbursement. Sven G. Meuth reports a relationship with Roche that includes: funding grants, speaking and lecture fees, and travel reimbursement. Sven G. Meuth reports a relationship with Sanofi-Aventis that includes: speaking and lecture fees and travel reimbursement. Sven G. Meuth reports a relationship with Springer Medizin Verlag that includes: speaking and lecture fees and travel reimbursement. Sven G. Meuth reports a relationship with STADA that includes: speaking and lecture fees and travel reimbursement. Sven G. Meuth reports a relationship with Chugai Pharma that includes: speaking and lecture fees and travel reimbursement. Sven G. Meuth reports a relationship with Teva that includes: speaking and lecture fees and travel reimbursement. Sven G. Meuth reports a relationship with UCB that includes: speaking and lecture fees and travel reimbursement. Sven G. Meuth reports a relationship with Viatris that includes: speaking and lecture fees and travel reimbursement. Sven G. Meuth reports a relationship with Wings for Life International that includes: speaking and lecture fees and travel reimbursement. Sven G. Meuth reports a relationship with Xcenda that includes: speaking and lecture fees and travel reimbursement. Frauke Zipp reports a relationship with Biogen that includes: consulting or advisory and funding grants. Frauke Zipp reports a relationship with Bristol Myers Squibb that includes: funding grants. Frauke Zipp reports a relationship with Janssen that includes: consulting or advisory. Frauke Zipp reports a relationship with Merck Serono that includes: consulting or advisory. Frauke Zipp reports a relationship with Novartis that includes: consulting or advisory. Frauke Zipp reports a relationship with Roche that includes: consulting or advisory. Frauke Zipp reports a relationship with Sanofi Genzyme that includes: consulting or advisory. Frauke Zipp reports a relationship with Sandoz that includes: consulting or advisory. Vinzenz Fleischer reports a relationship with Novartis that includes: funding grants. Stefan Bittner reports a relationship with Biogen Idec that includes: speaking and lecture fees. Stefan Bittner reports a relationship with Bristol Myers Squibb that includes: speaking and lecture fees. Stefan Bittner reports a relationship with Hexal that includes: speaking and lecture fees. Stefan Bittner reports a relationship with Merck Healthcare that includes: speaking and lecture fees. Stefan Bittner reports a relationship with Mylan that includes: speaking and lecture fees. Stefan Bittner reports a relationship with Novartis that includes: speaking and lecture fees. Stefan Bittner reports a relationship with Roche that includes: speaking and lecture fees. Stefan Bittner reports a relationship with Sanofi Genzyme that includes: speaking and lecture fees. Stefan Bittner reports a relationship with Teva that includes: speaking and lecture fees. If there are other authors, they declare that they have no known competing financial interests or personal relationships that could have appeared to influence the work reported in this paper.
