# Supplementary material for: Treatment approaches to patients with multiple sclerosis and coexisting psoriasis – A longitudinal multicenter observational cohort study
Source: Neurotherapeutics. 2026 Apr 29;23(3):e00914. doi: 10.1016/j.neurot.2026.e00914 (PMC13141705; doi:10.1016/j.neurot.2026.e00914)
Supplement: Multimedia component 1 [file mmc1.docx]

**Supplementary Tables**

**Table 1:**

|  | **PsO (n=32)** | **PsA (n=6)** |
| --- | --- | --- |
| **Age (years) [mean, SD]** | 45.4 ± 11.9 | 43.4 ± 10.6 |
| **Age at diagnosis (years) [mean, SD]** | 37.6 ± 11.7 | 36.0 ± 11.4 |
| **Age at first event (years) [mean, SD]** | 34.9 ± 11.6 | 35.2 ± 10.3 |
| **Sex (female) [%]** | 21 (66.0) | 3 (50.0) |
| **Disease duration (years) [mean, SD]** | 9.3 ± 9.9 | 7.0 ± 6.3 |
| **Disease course, n (%)**  **RRMS**  **SPMS**  **PPMS** | 25 (78.1)  5 (15.6)  2 (6.3) | 6 (100)  0 (0)  0 (0) |
| **EDSS [mean, SD]** | 2.5 ± 2.2 | 3.0 ± 2.9 |
| **Current DMT, n (%)**  **None**  **Moderate**  **High**  **Other** | 4 (12.5)  10 (31.2)  14 (43.8)  4 (12.5) | 0 (0)  1 (16.6)  1 (16.6)  4 (66.7) |

**Baseline demographic and clinical characteristics of MS patients with psoriasis (MS+PsO) compared with MS patients with psoriasis arthritis (PsA).** **DMT:** disease-modifying therapy (**moderate:** interferons, glatiramer acetate, fumarates, teriflunomide; **high:** natalizumab, anti-CD20 antibodies, cladribine, sphingosine-1-phosphate receptor modulators; **other:** azathioprine, methotrexate, anti-IL-17A antibodies, sulfasalazine). **EDSS:** Expanded Disability Status Scale; **MS:** multiple sclerosis; **PsO:** psoriasis; **RRMS:** relapsing–remitting multiple sclerosis; **SPMS:** secondary progressive multiple sclerosis; **PPMS:** primary progressive multiple sclerosis.

**Table 2:**

|  | **MS+PsO (n=38)** | **MS+IBD/RA (n=22)** | ***adj.-p-value^c^*** |
| --- | --- | --- | --- |
| **Age (years) [mean, SD]** | 45.4 ± 11.9 | 42.8 ± 16.5 | *>0.9999^a^* |
| **Age at diagnosis (years) [mean, SD]** | 37.6 ± 11.7 | 35.5 ± 11.8 | *0.7482^a^* |
| **Age at first event (years) [mean, SD]** | 34.9 ± 10.5 | 31.1 ± 7.5 | *0.7660^a^* |
| **Sex (female) [%]** | 24 (63.2) | *18 (81.8)* | *>0.9999^b^* |
| **Disease duration (years) [mean, SD]** | 9.3 ± 9.9 | 8.7 ± 8.3 | *>0.9999^a^* |
| **Disease course, n (%)**  **RRMS**  **SPMS**  **PPMS** | 31 (82)  5 (13)  2 (5) | 19 (86.4)  3 (13.6)  0 (0) | *>0.9999^b^* |
| **EDSS [mean, SD]** | 2.5 ± 2.2 | 2.8 ± 2.6 | *0.9602^a^* |
| **Current DMT, n (%)**  **None**  **Moderate**  **High**  **Other** | 4 (11)  12 (32)  14 (37)  8 (21) | *6 (27.3)*  *4 (18.2)*  *9 (40.9)*  *3 (13.6)* | *0.8605^b^* |

**Baseline demographic and clinical characteristics of MS patients with psoriasis (MS+PsO) compared with MS patients with other autoimmune comorbidities (inflammatory bowel disease or rheumatoid arthritis; MS+IBD/RA).** **Statistical tests: a**: Mann–Whitney U test; **b:** Fisher’s exact test or chi-square test; **c:** FDR correction with the Benjamini–Hochberg method. **DMT:** disease-modifying therapy (**moderate:** interferons, glatiramer acetate, fumarates, teriflunomide; **high:** natalizumab, anti-CD20 antibodies, cladribine, sphingosine-1-phosphate receptor modulators; **other:** azathioprine, methotrexate, anti-IL-17A antibodies, sulfasalazine). **EDSS:** Expanded Disability Status Scale; **IBD:** inflammatory bowel disease; **MS:** multiple sclerosis; **PsO:** psoriasis; **RA:** rheumatoid arthritis; **RRMS:** relapsing–remitting multiple sclerosis; **SPMS:** secondary progressive multiple sclerosis; **PPMS:** primary progressive multiple sclerosis.

| **DMT** | **MS only (n)** | **MS worsening, n (%)** | **Age (mean + SD)** | **Female, n (%)** | **MS/PsO (n)** | **MS worsening*, n (%)** | **Age (mean + SD)** | **Female, n (%)** |
| --- | --- | --- | --- | --- | --- | --- | --- | --- |
| **INF** | 75 | 35 (46.7) | 37.7 ± 11.5 | 49 (65.3) | 8 | 4 (50.0) | 35.5 ± 10.2 | 6 (75.0) |
| **GLAT** | 57 | 41 (71.9) | 38.1 ± 10.6 | 39 (68.4) | 11 | 9 (81.8) | 38.9 ± 9.6 | 9 (81.8) |
| **DMF** | 41 | 11 (26.8) | 36.0 ± 11.6 | 29 (70.7) | 26 | 6 (23.1) | 37.8 ± 11.5 | 20 (77.0) |
| **CD20** | 16 | 2 (12.5) | 42.6 ± 9.9 | 10 (62.5) | 9 | 1 (11.1) | 45.0 ± 10.7 | 5 (56.0) |

**Table 3:**

**MS worsening across DMTs in MS-only and MS/PsO cohorts: CD20:** anti-CD20 antibodies; **DMF:** dimethyl fumarate; **GLAT:** glatiramer acetate; **INF:** interferons; **MS:** multiple sclerosis; **PsO:** psoriasis; **SD:** standard deviation. *****MS-worsening and worsening of MS+PsO combined.

| **DMT (n)** | **Age at DMT (mean ± SD)** | **Female, n (%)** | **RRMS, n (%)** | **PMS, n (%)** | **EDSS (mean ± SD) at DMT** |
| --- | --- | --- | --- | --- | --- |
| **DMF (26)** | 37.8 ± 11.5 | 20 (77.0) | 26 (100) | 0 (0.0) | 1.1 ± 1.2 |
| **INF (8)** | 35.5 ± 10.2 | 6 (75.0) | 6 (75.0) | 2 (25.0) | 1.8 ± 1.2 |
| **GLAT (11)** | 38.9 ± 9.6 | 9 (81.8) | 10 (91.0) | 1 (9.0) | 1.8 ± 1.4 |
| **TER (4)** | 53.8 ± 11.1 | 0 (0) | 4 (100) | 0 (0.0) | 2.3 ± 0.3 |
| **S1P (5)** | 42.2 ± 6.5 | 3 (60.0) | 3 (60.0) | 2 (40.0) | 4.2 ± 2.0 |
| **NTZ (4)** | 31.5 ± 6.5 | 3 (60.0) | 4 (100) | 0 (0.0) | 1.3 ± 0.7 |
| **CD20 (9)** | 45.0 ± 10.7 | 5 (56.0) | 7 (78.0) | 2 (8.0) | 3.7 ± 2.2 |
| **IL17 (9)** | 38.3 ± 15.7 | 5 (44.4) | 8 (89.0) | 1 (11.0) | 1.3 ± 1.5 |

**Table 4:**

**Baseline characteristics for each DMT in the MS/PsO cohort: CD20:** anti-CD20 antibodies; **DMF:** dimethyl fumarate; **DMT:** disease modifying therapy; **EDSS:** Expanded disability status scale; **GLAT:** glatiramer acetate; **INF:** interferons; **IL17:** interleukin-17A antibodies; **NTZ:** natalizumab **PMS:** progressive multiple sclerosis; **RRMS:** relapsing–remitting multiple sclerosis; **SD:** standard deviation; **S1P:** sphingosine-1-phosphate (S1P) modulators; **TER:** teriflunomide.
